# Flavone Enhances Dengue Virus Type-2 (NGC Strain) Infectivity and Replication in Vero Cells

**DOI:** 10.3390/molecules17032437

**Published:** 2012-02-28

**Authors:** Keivan Zandi, Rafidah Lani, Pooi-Fong Wong, Boon-Teong Teoh, Sing-Sin Sam, Jefree Johari, Mohd Rais Mustafa, Sazaly AbuBakar

**Affiliations:** 1 Tropical Infectious Diseases Research and Education Center (TIDREC), Department of Medical Microbiology, Faculty of Medicine, University Malaya, Kuala Lumpur 50603, Malaysia; Email: keivan@um.edu.my (K.Z.); rafidahpoh@yahoo.com (R.L.); boonlim82@yahoo.com (B.-T.T.); singsin83@yahoo.com (S.-S.S.); jefree.johari@hotmail.com (J.J.); 2 Department of Pharmacology, Faculty of Medicine, University Malaya, Kuala Lumpur 50603, Malaysia; Email: wongpf@um.edu.my (P.-F.W.); rais@um.edu.my (M.R.M.)

**Keywords:** antiviral, dengue, flavones, flavonoid, molecule

## Abstract

This study investigates the effects of 2-phenyl-1-benzopyran-4-one (flavone) on DENV-2 infectivity in Vero cells. Virus adsorption and attachment and intracellular virus replication were investigated using a foci forming unit assay (FFUA) and quantitative RT-PCR, respectively. Addition of flavone (100 μg/mL) significantly increased the number of DENV-2 foci by 35.66% ± 1.52 and 49.66% ± 2.51 when added during and after virus adsorption to the Vero cells, respectively. The average foci size after 4 days of infection increased by 33% ± 2.11 and 89% ± 2.13. The DENV-2 specific RNA copy number in the flavone-treated infected cells increased by 6.41- and 23.1-fold when compared to the mock-treated infected cells. Flavone (100 μg/mL) did not promote or inhibit Vero cell proliferation. The CC_50_ value of flavone against Vero cells was 446 µg/mL. These results suggest that flavone might enhance dengue virus replication by acting antagonistically towards flavonoids known to inhibit dengue virus replication.

## 1. Introduction

Dengue virus (DENV) is a mosquito-borne virus belonging to the *Flaviviridae* family. There are four genotypes of dengue viruses and all four can cause dengue fever and its more severe forms dengue hemorrhagic fever and dengue shock syndrome [1]. Dengue causes significant health and economic burden to many tropical countries. Increasing urbanization, population growth and population mobility has contributed to further expansion of dengue-affected zones beyond the tropical and subtropical regions. The dengue quandary is further compounded by the lack of vaccines and antiviral drugs for effective prevention and control of the disease. Hence, there remains a need to intensify research to identify and develop potential antiviral drugs and vaccines for dengue.

Phytochemicals remain the focus of many *in vitro* studies seeking compounds specific against dengue [2,3,4]. Among the important phytochemicals, flavonoids, which are polyphenolic compounds, have attracted great interest from the scientific community because of their diverse biological properties such as anticancer, antioxidant, and antimicrobial (including antiviral) activities [5,6,7,8,9,10]. Several studies on the effects of various bioflavonoids on dengue virus replication efficiency have been reported [11,12]. Flavonoids such as baicalein, apigenin, and fisetin have been shown to exert antiviral effects on hepatitis B, influenza viruses and dengue virus [11,12,13,14,15]. There are also reports of inhibition of the virus replication cycle by flavones, a subgroup of flavonoids that include compounds such as wogonin, apigenin and luteolin [9,14,15]. These compounds share a common 2-phenylchromen-4-one (2-phenyl-1-benzopyran-4-one) backbone. To date, there have been no reports on the effects of flavone itself on dengue virus replication. In the present study, we investigate the effects of the flavone backbone on DENV-2 replication.

## 2. Results and Discussion

### 2.1. Cytotoxicity of Flavone against Vero Cells

African green monkey kidney cells (Vero) were treated with flavone for 4 days. The four day treatment duration was also the length of time used for evaluation of its activity on dengue virus replication. The cytotoxicity effects of flavone on Vero cells were determined using a MTT assay performed as previously described [16]. The CC_50_ value of flavones was 446 µg/mL when added directly to the cells. It was determined from this assay that 100 μg/mL flavone exerted no significant effects on cell viability and this concentration was used for all the subsequent studies. There was no observed cytotoxicity for cells treated with 1% DMSO, the solvent used to initially dissolve flavone. 

### 2.2. Evaluation of the Effects of Flavone on DENV-2 Replication

**Figure 1:**
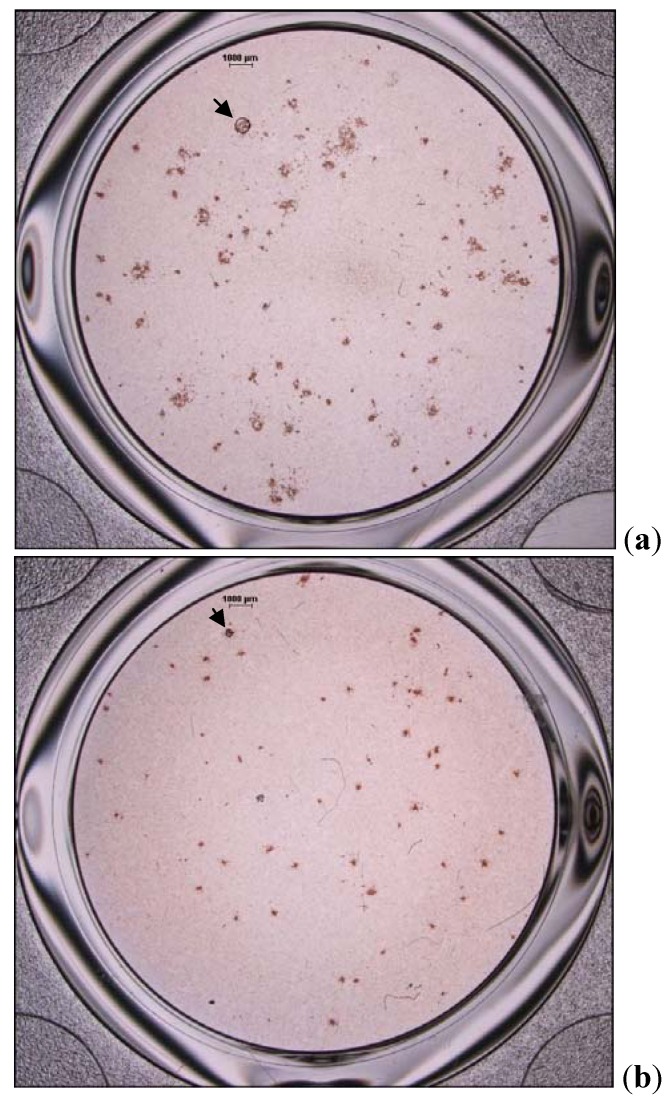
The effects of flavone on DENV-2 foci formation in Vero cells. Infected cells were treated with 100 µg/mL of flavone (**a**) or mock-treatment medium (**b**) during virus adsorption and attachment. Virus foci were detected after 4 days of incubation using dengue hyperimmune sera. Enlarged viral foci diameter was obvious in the flavone-treated cells (arrows).

**Figure 2:**
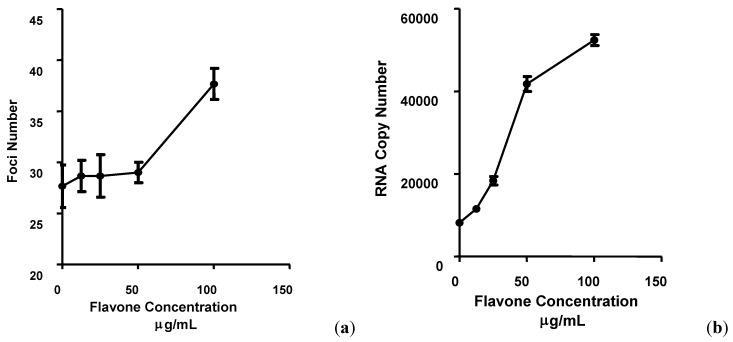
The effects of flavone on DENV-2 infection of Vero cells. Cells were treated with flavone during virus adsorption and the number of foci formed at 4 days post-infection was determined using the foci forming unit assay (**a**) and the respective DENV-2 specific RNA copy number was quantified using qRT-PCR (**b**). All experiments were performed in triplicates. Data (n = 3) were plotted using Graph Pad Prism Version 5.

The effects of flavone on adsorption and attachment of DENV-2 to Vero cells were determined by measuring the foci size and determining the number of viral foci formed on day 4 post-infection (PI) (Figure 1). In the presence of 100 µg/mL of flavone; added during virus adsorption and continuously after virus adsorption, the size of the viral foci on average increased by 33% ± 2.11 and 89% ± 2.13, respectively (Figure 2a). 

**Figure 3:**
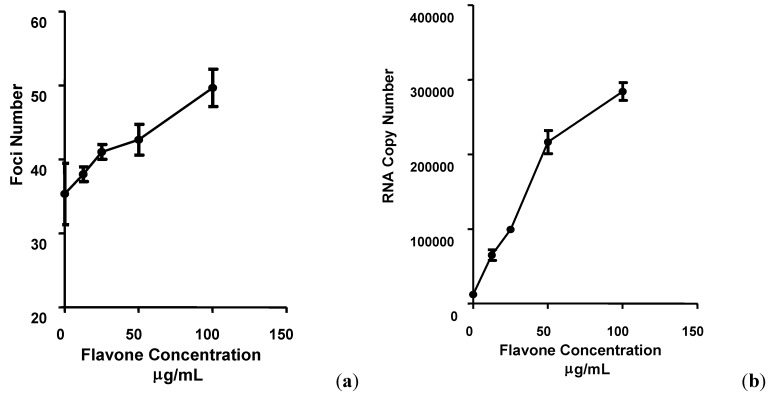
The effects of flavone on intracellular DENV-2 replication in Vero cells. Cells were treated with flavone continuously after virus adsorption and the number of foci formed was determined using the foci forming unit assay (**a**) and the respective DENV-2 RNA copy number was quantified using qRT-PCR (**b**). All experiments were performed in triplicates. Data (n = 3) were plotted using Graph Pad Prism Version 5.

The number of DENV-2 foci in Vero cells treated with 100 µg/mL of flavone at the time of virus adsorption and continuously after adsorption increased by 35.66% ± 1.52 and 49.66% ± 2.51, respectively, compared to the non-treated infected cells (Figure 3a). DENV-2 RNA copy number for cells treated with 100 µg/mL of flavone at the time of virus adsorption or after the infection was 6.41 and 23.1 times higher than the copy number of viral RNA from cells infected in the absence of flavone, respectively (Figure 2b and Figure 3b). These results suggest that DENV-2 infectivity was enhanced in cells treated with flavone, perhaps resulting in increased number of cells bearing progeny virus after a single round of infection. The increase in the size and number of foci was corroborated by the increased viral RNA copy number, suggesting possible enhancement of intracellular DENV-2 replication. A number of previous studies have identified antiviral activities against some DNA or RNA viruses in various flavonoids as well as flavones such as baicalein [1,7,9,10,11,12,13]. 

There are, however, no published reports on the enhancement of virus replication by flavones. Drugs such as the pentavalent antimony compound sodium stibogluconate, arsenic trioxide and carbonyl cyanide *m-*chlorophenylhydrazone, on the other hand, have been shown to enhance HIV-1 replication [17,18]. The effects on replication enhancement of HIV-1 is suggested to involve intracellular signaling pathways such as the mitogen activated protein kinase/extracellular signal-related kinase signal-transduction pathways and the activation nuclear factor—κB and activator protein 1, known to regulate viral transcription [17]. In the case of arsenic trioxide it was thought that the compound counteracted the reverse transcription block caused by human Ref1. The mechanisms of how flavone enhances dengue virus infection on the other hand, are unknown. Results from studies with HIV, however, highlighted a few possible mechanisms and these include probable antagonistic effects on viral replication suppression by interferon. It is also worth noting that while some flavonoids inhibit dengue virus replication [11,12], flavone, which shares a common backbone with those flavonoids, produced the opposite effects. These results strongly suggest the possible involvement of a common regulatory pathway or protein affecting dengue virus replication that is affected by these compounds, albeit antagonistically. Future studies will be needed to address this possibility. 

## 3. Experimental

### 3.1. Chemicals

Flavone was purchased from Sigma Chemical Company (Sigma, St. Louis, MO, USA). Stock flavone solution was prepared in dimethyl sulfoxide (DMSO) (40 mg/mL) and stored at −20 °C. For the study, the stock solution was serially diluted in serum free cell culture medium and sterile-filtered using syringe filter with 0.2 μm pore size. The highest final concentration of DMSO used to treat cells was at 0.5%.

### 3.2. Cells and Virus

Dengue virus type-2 (DENV-2) New Guinea C strain (NGC) was used for the study. Virus was propagated in C6/36 *Aedes albopictus* monolayer cells at 28 °C in the presence of 3% CO_2_ and harvested on day 7 PI. Virus stock was prepared and titrated as previously described [19] and stored at −80 °C until needed. African green monkey kidney cells (Vero) was used for the foci forming unit assay (FFUA). Cells were cultured in Eagle’s Minimum Essential Medium (EMEM) containing 10% fetal bovine serum (FBS, Gibco, NY, USA) at 37 °C in the presence of 5% CO_2_. For maintenance medium, serum concentration was reduced to 2%.

### 3.3. Cytotoxicity Assay

The cytotoxicity of flavone was determined using the 3*-*(4,5-dimethylthiazol-2-yl)*-*2,5-diphenyltetrazolium bromide MTT assay, as previously described [16]. Confluent Vero cell monolayers were treated in triplicate with increasing concentrations of flavone. The treated cells were incubated for 4 days at 37 °C. After 4 days, MTT solution (Promega, Madison, WI, USA) (15 µL) was added to all wells. The microplate was incubated at 37 °C for 4 h in a humidified 5% atmosphere. After the incubation period, solubilization/stop solution (100 µL) was added to the wells. The absorbance values of the wells were measured at 570 nm using a 96-well plate reader (TECAN, Mannendorf, Switzerland). 

### 3.4. Evaluation of the Effect of Flavone on DENV-2 Replication

The effects of flavone on DENV-2 adsorption and attachment to cells as well as its intracellular replication were determined by examining the changes in: (i) the average size of viral foci; (ii) number of viral foci formed and (iii) DENV-2 RNA copy number, as determined by quantitative RT-PCR. All experiments were performed in triplicate with different concentrations of flavone. Briefly, to determine the effects of flavone on virus adsorption, Vero cells monolayer was prepared in 24-well cell culture microplates and inoculated with 200 FFU of DENV-2 in the presence or absence of different concentrations of flavone. The microplates were incubated at 37 °C for 1 h for virus adsorption and attachment to the cells. The cells were then washed with sterile PBS twice and incubated at 37 °C for four days.

To evaluate the effects of flavone on DENV-2 intracellular replication, confluent Vero cells monolayer in 24 wells cell culture microplates were infected with 200 FFU of DENV-2 and kept at 37 °C for 1 h for virus adsorption and attachment. Cells were washed twice with sterile PBS in order to remove the unadsorbed viruses. The cells were overlaid with 1.5% carboxymethylcellulose (CMC) containing cell-growth medium supplemented with 2% FBS containing different concentrations of flavone and the plates were incubated at 37 °C for 4 days. The size and number of viral foci were determined as described below.

### 3.5. Foci Forming Unit Assay

DENV-2 was titrated by foci forming assay as described previously [11]. Briefly, a Vero cell monolayer was prepared in 24 wells cell culture microplate. After attaining ~80% confluency, growth medium was removed and the cells were infected by DENV-2 in the presence or absence of flavone under the conditions described earlier. The microplate was incubated at 37 °C for 1 h for virus adsorption and attachment. Cells were then washed with sterile PBS twice and incubated at 37 °C for four days to allowviral foci to form. To visualize the DENV-2 foci, the cell culture medium was discarded and cells were washed gently three times with phosphate buffer saline (PBS). Then, 10% paraformaldehyde was added to fix the cells for 30 min at room temperature (RT) followed by three times washing with PBS. NP40 1% (Sigma, St. Louis, MO, USA) was added to permeabilize the cell for 10 min at RT. Cells were washed three times with PBS and blocked with 3% skim milk solution prepared in PBS for 2 h at RT. After another three washings with PBS, the cells were incubated with dengue hyperimmune serum (produced in rabbit) diluted in 1:500 using 1% skim milk solution at 37 °C for 1 h. Cells were then washed three times with PBS and incubated with goat anti-rabbit IgG conjugated with horse-radish peroxidase (HRP) at final concentration of 1:250 in 1% skim milk solution (Sigma, St. Louis, MO, USA). Finally, 3'-diaminobenzidine (DAB) peroxidase substrate (Thermo Scientific Pierce, Rockford, IL, USA) was added to each well to stain the virus foci. Viral foci were counted under a SMZ 1000 stereomicroscope (Nikon, Tokyo, Japan) and expressed as Foci-Forming-Unit (FFU). The foci size was measured with NIS-Elements D Software v3.0 (Nikon). 

### 3.6. Quantitative Real-Time Polymerase Chain Reaction (qRT-PCR)

The quantitative RT-PCR for determining DENV RNA copy number was performed as previously described [11,19]. Briefly, DENV-2 RNA was extracted from the DENV-2 infected cells and cell culture supernatant using the RNA extraction kit (Qiagen, Hilden, Germany). Quantitative RT-PCR was performed using SensiMix SYBR green mixture (Quantace, Watford, UK) in a total reaction volume of 20 µL consisting of ddH2O (7.4 µL), 2× SensiMix One-Step (10 µL), 50× SYBR Green solution (0.4 µL), RNase Inhibitor (10 units), 50 pmol each of forward (DNF) and reverse (D2R) primers, and the extracted DENV RNA (1 µL).

All amplifications were performed in triplicates using the DNA Engine Opticon system (MJ Research/Bio-Rad, Hercules, CA, USA) with following amplification cycles: reverse transcription at 50 °C for 30 min, initial denaturation at 95 °C (10 min), followed by 45 cycles of 95 °C for 15 s, 59 °C for 30 s and 72 °C for 30 s. Melting curve analysis was performed at temperature from 60 °C to 98 °C to verify the assay specificity (as previously described). For absolute quantitation of the viral RNA, a standard curve was established with a serially diluted RNA extracted from virus inoculum with known infectious virus titer.

### 3.7. Statistical Analysis

Statistical analysis was performed using Graph Pad Prism for Windows, version 5 (Graph Pad Software Inc., San Diego, CA, USA, 2005). 

## 4. Conclusions

Results from this study suggest that the addition of flavone enhances DENV-2 replication in Vero cells. The mechanisms of how flavones influenced dengue virus replication is not known. However, the virus replication enhancement effects are most prominent when the infected cells were treated with flavone after virus adsorption and attachment. This implies that flavone acts intracellularly on the infected cells. Since a number of flavonoids have previously been shown to inhibit dengue virus replication, it is likely that flavone works through a similar molecule or pathway affected by the flavonoids. Further study will be needed to examine this possibility. 
